# Enhancing pH Modulation and Calcium Ions Release in External Resorption Artificial Defects: A Comparative Analysis of Ultrasonic Activation Effects on Ca(OH)_2_, MTA, and CEM Cement

**DOI:** 10.1155/2024/8850548

**Published:** 2024-11-14

**Authors:** Azadeh Kheradyar, Mamak Adel, Majid Sirati-Sabet, Alireza Kolahdouzan, Sahar Shafagh

**Affiliations:** ^1^Department of Endodontics, School of Dentistry, Tehran University of Medical Science, Tehran, Iran; ^2^Department of Endodontics, Dental Caries Prevention Research Center, Qasvin University of Medical Science, Qasvin, Iran; ^3^Department of Clinical Biochemistry, School of Medicine, Shahid Beheshti University of Medical Science, Tehran, Iran; ^4^Private Practice, Iran; ^5^Department of Endodontics, School of Dentistry, Qasvin University of Medical Science, Qasvin, Iran

**Keywords:** Ca(OH)_2_, CEM cement, external root resorption, MTA, ultrasonic energy

## Abstract

**Background and Objectives:** Diffusion of hydroxide (OH)^−^ and calcium (Ca)^++^ ions through dentin may cease external root resorption. Calcium hydroxide (Ca(OH)_2_), mineral trioxide aggregate (MTA), and calcium-enriched mixture (CEM) cement are the choices for this purpose due to their optimal properties. This study sought to analyze the effects of ultrasonic activation (UA) on pH and the release of calcium ions from Ca(OH)_2_, MTA, and CEM cement in external root resorption artificial defects.

**Materials and Methods:** This in vitro research involved the instrumentation and shaping of the root canals of 150 single-rooted teeth (#F4). External defects were intentionally made on the middle one-third of the root surface. Teeth were randomly assigned to a negative control group (*n* = 10), one positive control group (*n* = 20), and six experimental groups (each *n* = 20) according to the root canal filling material (Ca(OH)_2_ paste, MTA, CEM, gutta-percha, Ca(OH)_2_ paste+ultrasonic, MTA + ultrasonic, and CEM + ultrasonic). Ultrasonic energy was transferred to the test materials using a #25 spreader. Ca^++^ concentration and pH were measured after 1, 7, 15, and 30 days in all groups. Statistical analysis involved the use of repeated measure analysis of variance (ANOVA) and paired-sample *T*-test (*P* ≤ 0.05).

**Results:** The levels of calcium ions and pH increased significantly over time in all groups (*P* < 0.001). There were significant differences between the experimental groups in terms of pH and the concentration of released calcium ions at different times (*P* < 0.001). The Ca(OH)_2_ plus ultrasonic energy group ranked first, while the gutta-percha group ranked last in terms of release of calcium ions and pH.

**Conclusion:** The Ca(OH)_2_, MTA, and CEM cement groups showed an increase in pH and the release of calcium ions at the external root resorption defects. Additionally, the application of ultrasonic energy increased the release of calcium ions at these sites.

## 1. Introduction

External root resorption is a common complication in traumatized teeth, which occurs due to injury to the cementum layer covering the root surface following trauma or intracanal infection [1]. External root resorption may lead to serious consequences and eventual loss of the tooth [2]. The release of hydroxyl ions increases the pH level of dentin and the external root surface. The majority of microorganisms are destroyed at pH levels over 9.5 [3, 4]. A rise in pH toward alkalinity not only hinders the ability of osteoclasts to invade and function on the root surface but also creates a favorable condition for the formation of hard tissue [5, 6]. However, considering the buffering capacity of dentin, the diffusion of hydroxyl ions is limited in the external root surface, and a reduction in pH occurs compared to the internal root surface [7]. Calcium ions (Ca^++^) are important for the treatment of external root resorption defects. Calcium ions are responsible for the activation of adenosine triphosphate, which is necessary for the movement, specialization, and mineralization of osteoblasts. Then, the calcium ions that are released react with carbon dioxide in the tissues to produce calcium carbonate, an essential element for tissue remineralization [8, 9].

Calcium hydroxide (CH) is used for the treatment of external root resorption defects as an intracanal medicament [2]. CH releases hydroxyl and calcium ions and makes the environment alkaline [10]. Disadvantages of CH include decreasing root strength [2] and the need for several treatment sessions [2]. Studies have provided evidence that mineral trioxide aggregate (MTA) is a safe and biocompatible substance that can withstand oral fluids [11]. It has the ability to promote the growth of cementoblasts and odontoblasts, prevent root resorption, and create an effective seal [5]. Similar to CH, MTA also raises the pH level and exhibits antibacterial properties [11]. MTA is a calcium silicate-based material consisting of 50%–75% calcium oxide and 15%–25% silicon dioxide. These components, when combined, form compounds such as tricalcium silicate, dicalcium silicate, tricalcium aluminate, and tetracalcium aluminoferrite [12].

The calcium-enriched mixture (CEM) cement was introduced as an alternative to MTA. It has clinical applications in root canal therapy similar to those of MTA but with a different chemical composition. The major components of CEM cement are CaO, SO_3_, P_2_O_5_, and SiO_2_, and minor components are Al_2_O_3_, Na_2_O, MgO, and Cl [13]. This cement creates an alkaline pH and has antimicrobial properties [14]. It has been shown that periodontal ligament regeneration and cementogenesis occur in the presence of CEM cement [15].

Ultrasonic systems are extensively used in endodontics. Ultrasonic vibrations decrease the friction between cement particles and yield an unstable cement compound. This leads to the liberation and subsequent spread of hydroxyl and calcium ions into the tubules of the dentin and the external surface of the root. Thus, the pH levels and calcium ion concentrations increase in the external root surface, which may enhance the healing of resorption defects [16]. There is limited information available regarding the impact of ultrasonic activation (UA) on the levels of calcium ions and pH in external root resorption. In this regard, Duarte et al. [16] found a significant effect of UA on Ca ions and pH using Ca (OH)_2_, while Marciano et al. [17] found UA did not significantly alter pH between the groups when using MTA. The purpose of this study was to assess the impact of UA on the pH and calcium ion release from CH, MTA, and CEM cement in external root resorption defects.

## 2. Materials and Methods

### 2.1. Collection of Samples

This experimental in vitro study involved 150 human permanent single-rooted teeth that had round cross-sections and a curvature of less than 10° [18]. The teeth were immersed in 5.25% sodium hypochlorite solution for 30 min. The teeth were washed with distilled water and then kept in a 0.9% saline solution for storage at room temperature until the experiment [19]. Periapical radiographs were obtained of the teeth in mesiodistal and buccolingual dimensions. The teeth with open apices, resorption, canal space calcification, caries, fracture, or root cracks were excluded.

### 2.2. Tooth Preparation

In order to standardize the samples, the tooth crowns were trimmed using a high-speed handpiece and a diamond fissure bur (Tizkavan, Iran) to leave a remaining root length of 10 ± 1 mm [10]. A #15 K-file (Mani, Japan) was inserted into the canal until the tip of the file was visible at the apical foramen under a 10× magnification stereomicroscope (ZSM-1001, China). The working length was then determined by subtracting 1 mm from the determined file length. The teeth in which a #25 or smaller K file was not bound in the apical third of the roots were excluded. The ProTaper rotary system (Dentsply, Switzerland) was used to instrument the root canals in a single length technique. ProTaper rotary nickel-titanium files were used to the master apical file (MAF) of F4 (S1–S2–F1–F2–F3 and F4) operating at 300 rpm and 183 g/cm torque with an Endo IT (VDW, Germany). Once the preparation was finished, the root canals were flushed with 17% ethylenediaminetetraacetic acid (EDTA) (Ariadent; Asia Chemi Teb Co, Iran) to remove the smear layer and then rinsed for 1 min with 3cc of 5.25% sodium hypochlorite solution [2]. A final rinse with 5cc of saline was also performed. Root canals were then dried with sterile paper points (#45) (Ariadent; Asia Chemi Teb Co, Iran). In the middle third of the root, a cavity measuring 3 mm diameter and 1 mm of depth was prepared on the external buccal root surface by a high-speed handpiece and a #1 round diamond bur [10]. The entire diameter of the bur was embedded into the root surface to create these cavities. Smear layer removal of the created defects was done according to the protocol mentioned above. After that, nail polish was applied to the entire surface of the tooth, leaving out the areas with resorption defects. The teeth underwent digital radiography mesiodistally. The radiographs were evaluated in digital radiography software and the remaining dentin thickness in the floor of the resorption defects was measured by the software. To standardize the dentin thickness in the groups, samples with dentin thickness of 0.9–1.2 mm in the middle third of the root were entered in the study [20].

### 2.3. Grouping of Samples

The teeth were divided into eight groups, with one negative control group (*n* = 10), one positive control group (*n* = 20), and other six experimental groups (*n* = 20) assigned randomly as follows:

  Group CH: CH without ultrasonic energy. In this group, CH powder (Ariadent; Asia Chemi Teb Co, Tehran, Iran) was mixed with distilled water in a ratio of 0.6 mg/mL to obtain CH paste [21]. Afterward, a single millimeter of the CH paste was placed into the root canal with the use of an MTA carrier and condensed pluggers (Dentsply, Switzerland), respectively. This process was continued until 8 mm of the root canal length was filled.
  Group CH + U: CH plus ultrasonic energy. CH paste was prepared as described for the previous group with the exception that each 1 mm increment of CH paste was subjected to UA in three cycles of 20 s each using an ultrasonic unit with moderate power and E4D tip (NSK, NSK Ltd., Tokyo, Japan) to transfer ultrasonic energy to a size B spreader (Dentsply, Switzerland).
  Group M: MTA without ultrasonic energy. The ProRoot white MTA powder (Dentsply, Tulsa Dental, USA) was combined with distilled water in a 3:1 ratio as per the manufacturer's guidelines within this particular group [21]. The prepared paste was transferred into the root canal by an MTA carrier condensed by pluggers to condense 1 mm increments of MTA paste into the canal. This process was repeated until 8 mm of the root canal length was filled.
  Group M + U: MTA along with ultrasonic energy. MTA was prepared as in group M and ultrasonic energy was used as in group CH + U.
  Group C: CEM cement without ultrasonic energy. The CEM cement powder (BioniqueDent, Tehran, Iran) was mixed with liquid following the guidelines provided by the manufacturer. The prepared paste was transferred into the canal by an MTA carrier, and 1 mm increments were condensed by plugger. This process was continued until 8 mm of the canal length was filled.
  Group C + U: CEM cement with ultrasonic energy. CEM cement was prepared as in group C, and ultrasonic energy was used as in group CH + U.
  Group G (positive control group): The root canals in this set were filled using the lateral compaction technique with gutta-percha (Meta, Korea) and AH26 sealer (Dentsply, Germany).
  Group W (negative control group): Control group. Root canals in this group were filled with distilled water to the working length using an irrigation syringe.

To ensure successful filling of the root canals with the materials, radiographs with mesiodistal angulation were obtained. In case of the presence of voids, the materials were extracted via irrigation with saline, and the root canal was filled again, as explained earlier. The access cavity in the crown was filled with Cavit (Gholchai, Iran) and then sealed with a layer of sticky wax and two layers of nail polish to ensure a complete seal. Each tooth was placed in an Eppendorf plastic tube (capacity: 5cc) containing 5cc of phosphate buffer solution, which has a pH of 7.4. The composition of phosphate-buffered saline (PBS) includes NaCl, KCl, and PO_4_^3−^ in specific proportions to create a physiological medium, allowing the use of PBS solution in vitro to simulate the in vivo condition [22, 23]. The plastic tubes were stored in an incubator during the study period at 37°C under 100% humidity.

### 2.4. Measurement of pH and the Released Calcium Ions

The teeth were removed from the tubes at 1,7, 15, and 30 days, and the pH and concentration of calcium ions released into the solution were measured. At each measurement time point, 2cc of the 5cc saline in the Eppendorf tubes was extracted for measurement of the concentration of calcium ions, and the remaining solution was discarded. For further storage of teeth until the next assessment time point, phosphate buffer solution was added to the Eppendorf tubes [24]. To measure the pH, the teeth were washed with distilled water and then dried using sterile gauze. The cavity formed on the outer surface of the root was filled with distilled water, and the pH level was determined 3 min later using a digital pH meter (CHY, Taiwan) equipped with a microelectrode (Orion 2 star pH meter Thermo Electron Corporation, Waltham, MA, USA). Before each measurement, the microelectrode was calibrated using a solution with a known pH value [3]. To determine the amount of calcium, flame atomic absorption spectroscopy (flame-AAS) (GBS Avanta PM, Australia) was used. A diluting solution containing lanthanum nitrate was used to prevent interference from phosphate or alkaline compounds. Liquid samples were diluted in a 1:9 ratio, and calcium ion concentration was measured at a wavelength of 422.7 nm. A standard curve was generated using calcium carbonate salt at concentrations of 2, 4, 6, and 8 mg/L to determine the concentration of calcium ions in each sample.

### 2.5. Data Analysis

One-sample Kolmogorov–Smirnov test was used to assess the normal distribution of data regarding pH and concentration of calcium ions in different groups and at different time points. Tukey's honestly significant difference (HSD) test was used for pairwise comparison of groups. A pairwise comparison of pH and concentration of calcium ions at different time points was performed using the Bonferroni adjustment. Differences among the groups were analyzed using repeated measures analysis of variance (ANOVA) and paired *t*-test. The data were analyzed using SPSS version 20 (SPSS Inc., IL, USA). *P*  < 0.05 was considered statistically significant.

## 3. Results

### 3.1. pH

In this study, the pH and concentration of calcium ions in six experimental (*n* = 20), one positive control (*n* = 20), and one negative control (*n* = 10) groups were evaluated at 1, 7, 15, and 30 days. The mean pH of each group at each time point is shown in Table 1.

The pH increased over time in all groups (*P* < 0.001). The W group had the lowest pH at day 1 and the group CH + U had the highest pH at 30 days. The experimental groups had significant differences with each other in terms of alterations of pH (*P* ≤ 0.001).

Considering the interaction effect of time and group, one-way ANOVA was used for a more accurate comparison of groups. The findings indicated that the pH levels of the groups were significantly varied at all four time points (*P* < 0.001). Tukey's HSD test was used for pairwise comparison of groups (Table 2). Ultrasonic energy caused no significant difference in pH of CH, MTA, and CEM (*P*=0.95, *P*=0.99, and *P*=0.39, respectively). The pH of experimental groups only showed significant differences with that of groups G and W (*P* ≥ 0.001). Group CH + U had a significant difference with group C as well (*P* ≥ 0.001). Considering the significant interaction effect of time and groups, a pairwise comparison of time points within each group was also performed. In group CH, the pH on days 1 and 7 was not significantly different (*P*=0.56), but the difference in this regard between days 1 and 15, 1 and 30, 7 and 15, 7 and 30, and 15 and 30 was significant (*P* ≥ 0.001). In group G, the pH was not significantly different between days 1 and 7 (*P*=0.89), 1 and 15 (*P*=0.36), and 7 and 15 (*P* = 1). However, significant differences were noted between other time points (*P* ≥ 0.001). In group M, only the pH at days 1 and 7 was not significantly different (*P*=0.07), and significant differences were noted in pH at other time points (*P* ≤ 0.001).

### 3.2. Ca Ions

Table 3 shows the mean concentration of released calcium ions in each group at different time points.

As seen in Table 3, the W group had the least amount of calcium ion release on day 1, while the CH + U group had the highest release on day 30. The release of calcium ions significantly increased during the study period (*P* ≥ 0.001). The experimental groups showed significant variations in the pattern of changes in calcium ion concentration (*P* ≥ 0.001). However, the increase in calcium ion concentration in all groups was greater than the trend of increase in pH.

Pairwise comparison of groups for the calcium ion concentration by Tukey's HSD test revealed significant differences in the released calcium ions between groups CH and CH + U, groups M and M + U, and groups C and C + U (*P* ≥ 0.001). Pairwise comparisons showed significant variations in the amount of released calcium ions among the groups (*P* ≥ 0.001), with the exception of groups M and C + U (*P*=0.98) and groups G and W (*P*=0.95) (Table 4).

## 4. Discussion

External inflammatory resorption can cause tooth loss. The treatment of this condition is by changing the pH of the dentin to an alkaline level, interfering with the dissolving function of osteoclasts, and activating alkaline phosphatase to increase the tendency to repair external resorption [5]. During the setting process, the gradual release of calcium ions was observed in the tested materials [2]. The presence of calcium ions in the aqueous solution is necessary for the migration of osteoblasts. In addition, calcium increases the activity of remineralization in the external resorption area [16]. The present study utilized extracted human teeth to more accurately replicate the conditions found in the oral clinical setting. In contrast, Duarte et al. [16] extracted anterior bovine teeth, which possess distinct tubular and structural characteristics compared to human teeth. Cavities created on the external root surfaces had 1-mm depth and 3 mm of diameter and were standardized in all groups (based on the minimum penetration depth and diameter of the microelectrode). These dimensions were similar to previous studies [10, 19, 25]. However, considering the inverse correlation between dentin thickness and pH and level of calcium ions, in order to standardize the thickness of dentin as a confounder, only samples with 0.9–1.2 mm thickness of dentin remaining at the floor of the artificially created resorption defects were entered in the study [20]. The current results show that smear layer removal can open the dentinal tubules and increase the penetration of calcium and hydroxyl ions into the root dentin and root surface [16]. Foster, Kulild, and Weller [26] reported that smear layer removal enhanced the penetration of CH into the root canal dentin. Deardorf et al. [27] contradicted this claim and reported that EDTA absorbed calcium ions from the hydroxyapatite matrix, leading to the formation of calcium phosphate crystals. Due to high density, calcium phosphate crystals created a physical barrier and inhibited the diffusion of calcium ions [27]. In the current study, a microelectrode was used for pH measurement. It measures the amount of hydroxyl ions in small cavities. This method has high accuracy and has been used in many previous studies [10, 19]. Also, for the assessment of calcium ions, AAS was used. According to previous studies, AAS is utilized in cases where the concentration of calcium ions is below the detectable limit of conventional measurement methods. AAS enables precise quantification of calcium ion levels [16, 21, 25].

In the current research, significant differences were noted in the pH and concentration of calcium ions among the five groups of CH, MTA, CEM cement, gutta-percha, and distilled water. Over time, the pH and concentration of calcium ions increased in all experimental groups. The rise in pH showed a slower pattern in comparison to calcium ions, potentially attributed to the dentin's buffering capacity. Also, the results showed that the use of ultrasonic energy in all groups increased pH and release of calcium ions, but the increase in pH was not significant. However, ultrasonic energy significantly increased the release of calcium ions on the external root surface because dentin has no buffering capacity against calcium. There are fewer factors that can confuse the diffusion of calcium ions, and the use of ultrasonic energy can greatly improve the spread of this ion on the outer surface of the root. This finding is in line with the other results [3, 16, 25, 28]. Duarte et al. [16] reported a significant increase in the concentration of calcium ions over time and added that the use of ultrasonic energy, irrespective of the filling material used, increased the calcium ions at 7 and 30 days. The tested materials in their study included propylene glycol and distilled water, both with and without UA, with testing conducted at 7, 15, and 30 days [16]. George et al. [25] investigated 30 extracted anterior teeth divided into three groups: Group I, which served as a control with unfilled canals; Group II, where the root canal space was filled with MTA; and Group III, filled with ApexCal. The calcium ion release was measured at 1, 7, 12, 14, and 28 days. They reported that the increase in the release of calcium ions was not significant at day 1 but increased over time [25]. In a study by Ho et al. [3], the in vitro pH changes in root dentin over a 2-week period using 48 extracted bicuspids were investigated. The tested materials included Roeko CH plus points, aqueous CH paste, and gutta-percha points as a control. pH measurements were taken at 1, 2, 3 h, 1, 3, 7, and 14 days. They showed that the release of calcium ions over time significantly increased [3]. Silva and colleagues aimed to evaluate the influence of UA on the physicochemical properties of hydraulic calcium silicate-based sealers, specifically Bio-C Sealer, Sealer Plus BC, and Bio Root RCS. Nine experimental conditions were established based on the sealers and activation time (no activation, 10, and 20 s). The testing included assessments of initial and final setting times, flow, radiopacity, solubility, pH, and calcium ion release at 1, 24, 72, and 168 h. The study found that UA led to an increase in pH levels and the diffusion of calcium ions, resulting in improved healing in cases of root resorption [28]. Also, these findings regarding CH are in agreement with the study of Farhad et al. [21], which indicated an increase in the concentration of calcium ions and pH during their study period. Their methodology was similar to the current study and they also used AAS for measurement of calcium ions. De Vasconcelos et al. [29] demonstrated that the concentration of calcium ions released in the ProRoot MTA group increased over time at 24, 72, 36, and 168 h. They placed MTA paste in fine polyethylene tubes in deionized water (instead of root canals) but obtained results similar to ours [29]. Yazdanpanahi, Behzadi, and Jahromi [24] conducted a study to measure the pH changes at 1 and 2 weeks, and 1 and 3 months after applying CH and MTA. They found that the average pH was notably higher in the CH group at 1 and 2 weeks, which aligns with the present findings. However, in our study, this difference was not statistically significant. The discrepancy in the findings may be due to the use of various CH solvents in their research. Additionally, they reported that the mean pH level was significantly higher at 2 weeks compared to other time points, which may be due to the freshness of the solution after each measurement [24]. To express the advantages of increasing the calcium ions and pH level, a narrative review by Estrela et al. [30] showed CH, MTA, and calcium silicate cement can cause stimulation of mineralized tissue deposition because of their ability in Ca ions diffusion and increase pH level. Differences in the studies may be attributed to the environment in which the samples are stored, different methodologies (e.g., exchange of testing solution over time), different types of teeth, and variable thickness of residual dentin. The results showed pH in all experimental groups was between 6 and 10.5 at all time points. Thus, considering the fact that most microorganisms are destroyed at a pH over 9.5 [3, 4] and *Enterococcus faecalis* species can survive higher pH values (up to 11.5) [31], it may be concluded that even the CH + U with the maximum pH among the experimental groups cannot eliminate *E. faecalis* during 30 days; and as shown *E. faecalis* can cause failer in endodontic treatment [32]. However, other microorganisms can be destroyed by CH, CH + U, M, M + U, and C + U after 15–30 days. Moreover, the ideal pH for the activity of osteoclastic hydrolase is between 3.6 and 5 [33, 34]. Thus, all the study materials at all time points can activate this enzyme. Therefore, even a single session treatment with gutta-percha can activate this enzyme; however, in this state, bacteria are still present to activate osteoclasts and, subsequently, the process of resorption. The optimal pH for the activity of alkaline phosphates is 9.8 [35]. Thus, healing may be noted in group CH at 30 days, group CH + U at 15 and 30 days, and groups M and M + U at 30 days. Group G did not enhance healing. Further research is needed to evaluate the pH and release of calcium ions from intracanal medicaments, using longer study periods and other materials based on calcium silicate.

## 5. Conclusion

Within the limitations of this study, the pH and release of calcium ions increased during the 30-day study period in all groups. Ultrasonic energy did not significantly increase the pH at the artificially created external root resorption defects but significantly enhanced the release of calcium ions. The CH + U group exhibited the highest pH value and calcium ion release levels over the course of the experiment compared to the other groups.

## Figures and Tables

**Table 1:** Mean ± standard error of pH in different days and groups.

Day	CH	CH + U	M	M + U	C	C + U	G	W	*P*-value
1st day	8.92 ± 0.26	9.05 ± 0.14	8.58 ± 0.10	8.88 ± 0.10	8.16 ± 0.11	8.61 ± 0.18	7.13 ± 0.15	6.34 ± 0.15	<0.001
7th day	9.14 ± 0.21	9.34 ± 0.15	8.99 ± 0.14	9.23 ± 0.09	8.48 ± 0.12	8.89 ± 0.20	7.16 ± 0.15	6.78 ± 0.10	<0.001
15th day	9.53 ± 0.24	9.85 ± 0.15	9.52 ± 0.15	9.68 ± 0.11	8.84 ± 0.11	9.53 ± 0.22	7.19 ± 0.14	7.32 ± 0.12	<0.001
30th day	9.90 ± 0.24	10.25 ± 0.18	9.85 ± 0.15	10.00 ± 0.10	9.48 ± 0.15	9.96 ± 0.23	7.38 ± 0.15	7.78 ± 0.16	<0.001
*P*-value	<0.001	<0.001	<0.001	<0.001	<0.001	<0.001	<0.001	<0.001	—

*Note*: Time effect: *P* < 0.001, Group effect: *P* < 0.001, interaction time × group: *P* < 0.001.

Abbreviation: CH, calcium hydroxide.

**Table 2:** Results of Tukey HSD test for comparison of groups based on mean pH levels.

Main group	Other groups compared	Mean difference	*P*-value
CH	CH + U	−0.24	0.95
	M	0.08	1.0
	M + U	−0.09	1.0
	C	0.63	0.15
	C + U	0.12	0.99
	G	2.1	0.001
	W	2.7	0.001

CH + U	M	0.33	0.82
	M + U	0.15	0.99
	C	0.88	0.01
	C + U	0.37	0.75
	G	2.4	0.001
	W	2.9	0.001

M	M + U	−0.18	0.99
	C	0.54	0.31
	C + U	0.03	1.0
	G	2.0	0.001
	W	2.7	0.001

M+U	C	0.73	0.06
	C + U	0.22	0.97
	G	2.2	0.001
	W	2.8	0.001

C	C + U	−0.51	0.39
	G	1.5	0.001
	W	1.9	0.001

C+U	G	2.0	0.001
	W	2.6	0.001

G	W	1.5	1.0

Abbreviations: CH, calcium hydroxide; HSD, honestly significant difference.

**Table 3:** Mean ± standard error of calcium ions in different days and groups (mg/L).

Day	CH	CH + U	M	M + U	C	C + U	G	W	*P*-value
1st day	8.43 ± 0.33	10.28 ± 0.28	6.06 ± 0.12	6.42 ± 0.17	5.35 ± 0.13	5.49 ± 0.18	5.49 ± 0.20	5.23 ± 0.17	<0.001
7th day	9.71 ± 0.26	13.19 ± 0.28	8.86 ± 0.11	9.52 ± 0.23	7.40 ± 0.12	8.11 ± 0.23	6.11 ± 0.18	5.96 ± 0.18	<0.001
15th day	17.92 ± 0.23	19.60 ± 0.35	15.73 ± 0.10	17.46 ± 0.23	13.85 ± 0.11	14.72 ± 0.16	9.12 ± 0.27	8.88 ± 0.21	<0.001
30th day	26.61 ± 0.32	28.37 ± 0.27	21.91 ± 0.18	23.20 ± 0.26	20.46 ± 0.18	23.68 ± 0.28	11.94 ± 0.23	10.65 ± 0.20	<0.001
*P*-value	<0.001	<0.001	<0.001	<0.001	<0.001	<0.001	<0.001	<0.001	—

Abbreviation: CH, calcium hydroxide.

**Table 4:** Results of Tukey HSD test for comparison of groups based on mean calcium levels (mg/L).

Main group	Other groups compared	Mean difference	*P*-value
CH	CH + U	−2.1	0.001
	M	2.5	0.001
	M + U	1.5	0.001
	C	3.9	0.001
	C + U	2.6	0.001
	G	7.5	0.001
	W	7.9	0.001

CH + U	M	4.7	0.001
	M + U	3.7	0.001
	C	6.0	0.001
	C + U	4.8	0.001
	G	9.6	0.001
	W	10	0.001

M	M + U	−1.0	0.001
	C	1.3	0.001
	C + U	0.14	0.98
	G	4.9	0.001
	W	5.2	0.001

M + U	C	2.3	0.001
	C + U	1.1	0.001
	G	5.9	0.001
	W	6.3	0.001

C	C + U	−1.2	0.001
	G	3.6	0.001
	W	4.2	0.001

C + U	G	4.8	0.001
	W	5.2	0.001

G	W	2.1	0.95

Abbreviation: CH, calcium hydroxide.

## Data Availability

The data that support the findings of this study are available from the corresponding author upon reasonable request.
